# The Cutaneous Rabbit Illusion Affects Human Primary Sensory Cortex Somatotopically

**DOI:** 10.1371/journal.pbio.0040069

**Published:** 2006-02-28

**Authors:** Felix Blankenburg, Christian C Ruff, Ralf Deichmann, Geraint Rees, Jon Driver

**Affiliations:** **1**UCL Institute of Cognitive Neuroscience and Department of Psychology, University College London, London, United Kingdom; **2**Wellcome Department of Imaging Neuroscience, Institute of Neurology, University College London, London, United Kingdom; University of MinnesotaUnited States of America

## Abstract

We used functional magnetic resonance imaging (fMRI) to study neural correlates of a robust somatosensory illusion that can dissociate tactile perception from physical stimulation. Repeated rapid stimulation at the wrist, then near the elbow, can create the illusion of touches at intervening locations along the arm, as if a rabbit hopped along it. We examined brain activity in humans using fMRI, with improved spatial resolution, during this version of the classic cutaneous rabbit illusion. As compared with control stimulation at the same skin sites (but in a different order that did not induce the illusion), illusory sequences activated contralateral primary somatosensory cortex, at a somatotopic location corresponding to the filled-in illusory perception on the forearm. Moreover, the amplitude of this somatosensory activation was comparable to that for veridical stimulation including the intervening position on the arm. The illusion additionally activated areas of premotor and prefrontal cortex. These results provide direct evidence that illusory somatosensory percepts can affect primary somatosensory cortex in a manner that corresponds somatotopically to the illusory percept.

## Abstract 2

High resolution fMRI was used to demonstrate activity in primary somatosensory cortex in response to the illusion of a sensory stimulus.

## Introduction

Despite much general interest in studying the neural correlates of phenomenal perception, somatosensory perception has received relative little attention relative to other sensory modalities, such as vision. A major issue in the visual domain has been whether primary visual cortex activity is associated with visual awareness, as recently indicated [1,2]. Here we address an analogous issue for somatosensory cortex. To do so we exploited a robust somatosensory illusion [3], in combination with functional magnetic resonance imaging (fMRI) with improved spatial resolution at 3 tesla (T) in humans, while participants reported their phenomenal somatosensory experiences. We took advantage of the somatotopic nature of primary somatosensory cortex (SI), to assess whether activation there would reflect the physically stimulated locations of tactile stimuli on the skin, or instead might mirror their phenomenally perceived location, which differed from the physical location in the illusory situation.

The cutaneous rabbit (or somatosensory saltation) is a classic somatosensory illusion [3], whereby repetitive and rapid sequences of stimulation at two or more skin locations can, under certain conditions, lead to illusions that intervening space between the actual stimulation sites was stimulated on the body, as if a rabbit hopped along successive locations. Typically, when repeated stimulation at one site is followed by stimulation at a second site, some of the stimuli from the initial site are mislocalized in the direction of subsequent stimuli, thus providing an apparent example of perceptual postdiction [4] in the somatosensory system. Numerous behavioral studies [3,5,6] have identified behavioral constraints on this phenomenal illusion and its strength, such as the number, repetition, intensity, somatotopic location, and separation of stimuli.

It has been speculated based on indirect, purely behavioral grounds, that generation of the rabbit illusion may be cortical, and might conceivably even involve SI [7]. The illusion does not merely reflect peripheral interactions between stimulated skin sites, since it can be found even when skin areas between actual stimulation sites are anaesthetized [8]. Moreover, it reportedly cannot be induced across the body midline [8] without stimulation at the midline, in apparent congruence with contralateral representation in SI, with few if any transcallosal connections at this level [9]. Moreover, how far the rabbit can be made to jump along different regions of the body surface varies in approximate accord with receptive-field size for the corresponding SI neurons [7,8]. But there has previously been no direct evidence that the rabbit illusion can indeed affect activity in human SI, nor that activity there reflects phenomenal perceptions of where somatosensory stimuli appear to be located, rather than their actual physical location.

We employed a specially designed version of the cutaneous rabbit illusion, using electrical stimulation along the forearm as participants lay in the scanner. Sequences of stimulation near the wrist and elbow could, with appropriate timing parameters, lead to the illusion that this stimulation hopped along the forearm, evoking the feeling that intervening space along the forearm (between wrist and elbow) was stimulated in the sequence when in fact it was not. We used fMRI at 3T, with a custom magnetic resonance sequence to improve spatial resolution (see below). This allowed us to resolve somatotopic cortex for the forearm sufficiently to identify differential activation for three different skin sites along the forearm, by contrasting blocked functional localizers for these (which we examined at both a group and individual level). In the main experiment we assessed activation for stimulus sequences that either did include stimulation of the intervening skin site, or that illusorily felt as thought the intervening site had been stimulated (when in fact it had not), comparing both of these with control sequences that did not induce the illusion nor stimulated the intervening skin site.

**Figure 1:**
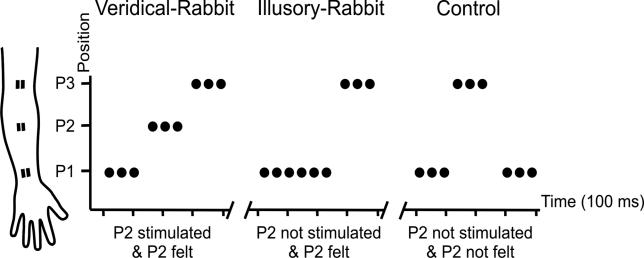
Schematics of the Stimulus Sequences Schematics of the stimulus sequences in the three main conditions (veridical-rabbit, illusory-rabbit, and control). The cartoon of the forearm schematically indicates the three different electrode positions (P1, P2, and P3). For the veridical-rabbit condition, three pulses at P1 were followed by three at P2 and then three at P3. The illusory-rabbit condition used a P1-P1-P3 sequence instead, but phenomenally, this was equivalent to the veridical-rabbit condition, with stimulation being felt around P2 for later repetitions at P1, despite no actual stimulation at P2. The control condition was a P1-P3-P1 sequence, thus stimulating the same two actual sites as for the illusory-rabbit condition, but now in a different sub-second order, which did not induce any illusion of stimulation around P2. The nine pulses in each condition were given in 400 ms.

Specifically, participants were stimulated with pulse trains that could form three distinct sequences (Figure 1). For the veridical-rabbit sequence (Figure 1), we stimulated three successive positions along the forearm in ascending order from wrist to elbow (P1-P2-P3). For the critical illusory-rabbit condition, pulses at the intervening position (P2) were substituted for by further pulses at the wrist (P1), to produce a P1-P1-P3 sequence (seeFigure 1). Importantly, this sequence produced the illusion that pulses were felt to hop along to the intervening location P2 before stimulation of P3 (i.e., some P1 pulses were mislocalized to P2). This made the veridical- and the illusory-rabbit sequences equivalent phenomenally, as confirmed with formal behavioral comparisons (see below). Veridical- and illusory-rabbit sequences thus differed in terms of whether P2 was actually stimulated or not, but did not differ in terms of whether any stimulation was felt to originate from P2. Finally, we also included control sequences P1-P3-P1 (Figure 1). These stimulated the same two skin sites as for the illusory-rabbit condition (i.e., P1 and P3), but now without inducing any illusion of stimulation at P2, due to the changed order within the rapid sequence.

This design enabled us to test whether the pattern of activity in somatotopic somatosensory cortex would reflect the similar phenomenological percept of touch at P2 that arose during both the illusory- and the veridical-rabbit sequences, or instead would follow the actual physical differences in stimulation for these conditions. We tested this by identifying brain regions that either showed common activity increases during both illusory- and veridical-rabbit stimulation, relative to the phenomenologically different control condition, or that displayed activity differences between the two rabbit conditions, commensurate with their physical differences. To allow somatotopic localisation of activity within somatosensory cortex, we also ran blocked localizer conditions, stimulating just P1, or P2, or P3 on the arm throughout each block (seeMaterials and Methods). In addition to assessing the data at a group level, we also analyzed individually defined somatotopic region of interest (ROI), based on the P1, P2, or P3 localizers, for each individual. Finally, in addition to examining somatosensory cortex in detail, we also analyzed the entire brain volume for any further differences (or commonalities) in neural activations for the illusory- and veridical-rabbit conditions. This might reveal higher-level brain areas potentially involved in the rabbit illusion, in addition to any activations in somatosensory cortex itself which was our main concern.

## Results

Inside the scanner, participants reported on each trial whether they did or did not feel stimulation at the intervening location, P2 (seeFigure 1 andMaterials and Methods). The rabbit illusion was robustly induced during scanning, with perception of P2 stimulation (despite its physical absence) reported on 90% of the illusory-rabbit trials. This did not differ from responses to veridical-rabbit trials, where participants correctly reported the presence of P2 stimulation on 93% of trials. Finally, participants correctly reported on 87% of the control trials that P2 stimulation was absent, confirming that no illusion was generated by stimulation of the same locations as for the illusory rabbit but in a different order (i.e., now P1-P3-P1 instead of P1-P1-P3; seeFigure 1). Behavioral response latencies did not differ significantly for the veridical-rabbit (442 ± 141 ms), illusory-rabbit (440 ± 150 ms), or control conditions (415 ± 130 ms).

**Figure 2:**
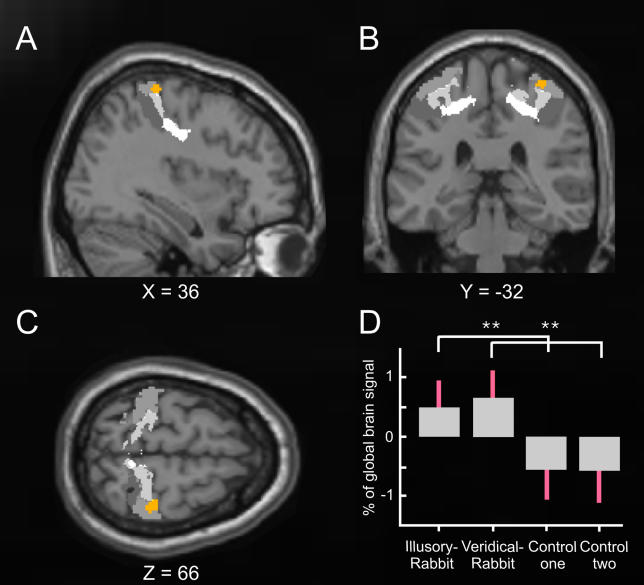
Common Activation for the Illusory-Rabbit and the Veridical-Rabbit versus Control Conditions Embedded in Probabilistic Cytoarchitectonical Maps of BAs 3a, 3b, 1, and 2 Statistical T-maps (orange) obtained from the random-effects group analysis for the conjunction contrast of illusory-rabbit versus control data, and of veridical-rabbit versus the other (equivalent but independent) control dataset. The activation in right SI (peak at X = 36, Y = −32, Z = 66,*p* <0.005 for display purposes) is projected on the (A) sagittal, (B) coronal, and (C) transverse slices of the Montreal Neurological Institute standard brain, superimposed on gray-level-coded cytoarchitectonical probability maps (BA 3a in very light grey, BA 3b in light grey, BA 1 in grey, and BA 2 in deep grey), taken from Eickhoff et al. (2005). The plot (D) shows the parameter estimates for the experimental conditions (standard errors indicated in red), extracted from the peak of the activation (orange) in right SI. Note that both the veridical-rabbit and the illusory-rabbit conditions showed significantly higher activation than the two control datasets (seeMaterials and Methods for why the latter were split to allow conjunction analysis), which were equivalent, whereas the two rabbit conditions did not differ from each other.

Hypothesis-driven analysis of the fMRI data sought to identify any commonalities or differences in activation of somatosensory cortex for the veridical-rabbit and the illusory-rabbit conditions. Stimulation at P2 was experienced for both of these sequences, even though P2 was not actually stimulated for the illusory condition. We therefore first determined whether there were any common activity increases within somatosensory cortex (considered as the postcentral gyrus and parietal operculum bilaterally) for the two rabbit conditions, relative to the control condition in which P2 was neither felt nor stimulated. This conjunction analysis [10] (seeMaterials and Methods) revealed that only one area, in right SI (i.e., right postcentral gyrus), contralateral to the stimulated arm, showed significantly higher activity during both the illusory- and veridical-rabbit sequences than during control sequences (Figure 2, activation shown in orange). Seventy-five percent of this activation was tentatively (but mechanistically) attributed to Brodmann Area (BA) 1 of SI using a three-dimensional probabilistic computerized cytoarchitectonic atlas, which is based on post-mortem data in normalized space [11] (see regions marked in gray inFigure 2, plus its legend).

Inspection of the level of activation evoked by the different experimental conditions in this region of SI confirms that it was indeed activated similarly and significantly by both the veridical-rabbit and the illusory-rabbit conditions, relative to the control (Figure 2D). This accords with the equivalent phenomenal percepts for the veridical- and illusory-rabbit conditions, despite their difference in actual stimulation (i.e., no P2 physical stimulation for the illusory rabbit, only its illusory perception). Note also that this joint activation by both rabbit conditions, relative to the control sequences, was highly specific, as we did not find any other activated areas within somatosensory cortex, not even when inspecting the single contrasts of veridical rabbit versus control, or of illusory rabbit versus control.

Having determined that the illusory rabbit activated contralateral SI, we next compared the location of this activation to the independently acquired somatosensory localizer data that mapped the somatotopic skin locations P1, P2, and P3 (seeMaterials and Methods). This revealed (seeFigure 3) that the location of the critical rabbit-related activation in contralateral somatosensory cortex (Figure 2) did indeed accord in a somatotopic fashion to the forearm region we termed P2, which was illusorily felt in the illusory-rabbit condition, but was actually stimulated only in the veridical-rabbit condition. The areas within SI responding most strongly to blocked stimulation of P1, P2 or P3 (in the localizer session) followed the expected medial-to-lateral topography (seeFigure 3). Crucially, the region activated by both rabbit conditions versus the control sequences (orange) fell in the centre of the localizer activation for P2 (seeFigure 3C) versus P1 and P3. As a further assessment of this, we took the critical rabbit-activation region (see orange inFigure 2 andFigure 3) and then extracted from this same region the mean activity produced by P1, P2, or P3 stimulation during the separate localizer runs (seeFigure 3D). This confirmed a reliably stronger response in this rabbit-related region from the main experiment, to P2 stimulation in the separate localizer runs than to stimulation of P3 (in 9/10 participants) or of P1 (in 8/10 participants) in the localizers. Thus, the experimental rabbit-related activation in contralateral somatosensory cortex fell in the somatotopically appropriate region corresponding to stimulation (or in the case of the illusory rabbit, illusory perception) at region P2 on the forearm (seeFigure 3).

**Figure 3:**
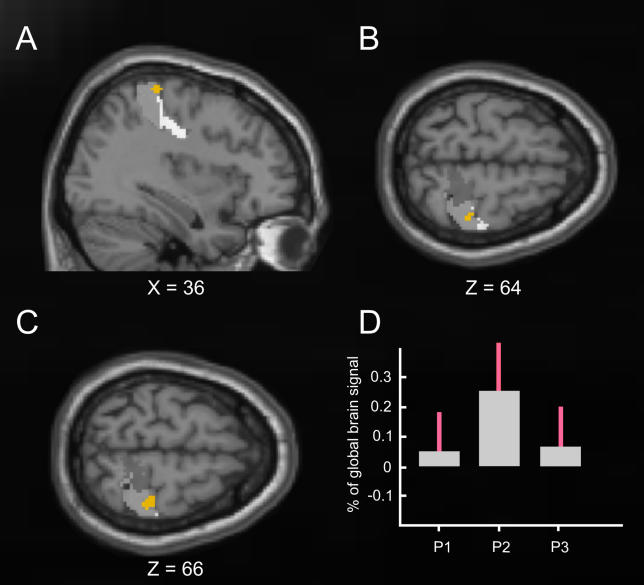
Common Activation for the Illusory-Rabbit and the Veridical-Rabbit versus Control Conditions Embedded in the Localizer Results for Skin-Sites P1, P2, and P3 The brain images (A, B, C) again show the common activation (random-effects group analysis) for the veridical-rabbit and illusory-rabbit conditions in orange (peak at X = 36, Y = −32, Z = 66), now projected onto the differential contrasts for the localizer conditions (P1 versus P2 and P3 in bright gray, P2 versus P1 and P3 in intermediate gray, P3 versus P1 and P2 in dark gray) within BAs 3a, 3b, 1, and 2, as defined by the cytoarchitectonic atlas [11]. The differential activations for P1, P2, and P3 show the expected medial-to-lateral ordering for the different forearm positions (B and C). Although there is some spread in these localizer activations (as expected for smoothed fMRI data across a group, but see alsoFigure 4), note that the critical experimental activation for the two rabbit conditions (shown in orange here) clearly falls quite centrally within the localizer activation that corresponds to P2 (see B and C). Moreover, (D) plots the parameter estimates (SPM beta-values and standard errors in red) extracted from the region that was experimentally activated by the rabbit (orange), showing these for each of the separate group-localizer conditions. Note the stronger response to P2 than P3 or P1 here, as shown by the vast majority of individuals (9/10 and 8/10 respectively, see main text).

**Figure 4:**
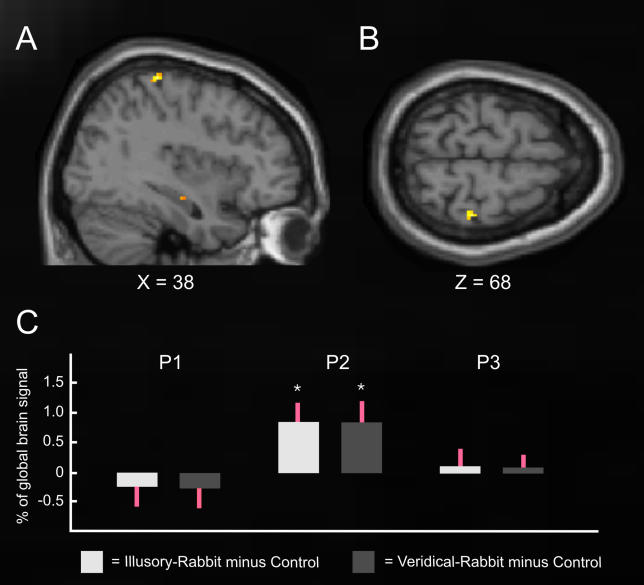
Common Activation for the Illusory-Rabbit and the Veridical-Rabbit versus Control Conditions with Less Spatial Smoothing The brain images (A, B) show the common activation for the veridical-rabbit and illusory-rabbit in yellow (*p* < 0.001, uncorrected), from a group analysis using a considerably reduced smoothing kernel (4-mm FWHM). Note that the activation elicited by both the illusory- and veridical-rabbit (relative to the control) conditions is in virtually the identical location within SI (peak at X = 38, Y = −32, Z = 68) as before (seeFigure 3), and at the same threshold. Panel C shows the outcome of single-participant ROI analysis of the mean parameter estimates (SPM beta-values, from the analysis with 4-mm FWHM smoothing), extracted separately for each individual from the participant-specific locations within SI responding maximally to stimulation at P1, P2, or P3 (relative to the other two skin sites from these three) during the individual localizer sessions. The bars show the signal change during illusory- (left bar in each pair) and veridical- (right bar in each pair) rabbit, relative to the control conditions (standard errors indicated in red), for the individually defined cortical ROIs activated by P1, P2, or P3 stimulation (see above). This individual analysis thus confirms that both rabbit conditions led to significant activity increases (relative to control) only within the individual participant-specific cortical representations of P2, but not of P1 or P3. Thus, the rabbit-related activations corresponded to the skin location where stimulation was illusorily felt in the critical rabbit condition.

We also repeated the group analysis but with considerably less spatial smoothing (4-mm, full-width, half-maximum [FWHM] instead of 9-mm FWHM, seeMaterials and Methods). The outcome (seeFigure 4A and4B) was virtually identical in terms of spatial localization and statistical significance. To provide a further detailed assessment, we also conducted single-participant ROI analyses of activity for the experimental conditions with the reduced smoothing, within each individual's own cortical representations of skin sites P1, P2, and P3, centering each ROI on participant-specific peaks from the contrasts of their own individual localizers (i.e., P2 versus P1-and-P3, etc; seeMaterials and Methods). From each such individually defined ROI (i.e., corresponding to P1, P2, or P3), we extracted the mean signal (parameter estimates) during the veridical- or illusory-rabbit conditions, relative to the control condition. This individually defined analysis (Figure 4C) revealed that both the illusory and the veridical rabbit led to significantly increased activity (both*p* < 0.025) only in the sector of SI that responded to P2 stimulation in the individual localizers, not in the representations of skin sites P1 and P3. This single-participant analysis thus further confirms that activity in the primary cortical representation of stimulation-site P2 (now determined independently for each individual, from their own localizer session) reflected the illusorily felt location during the rabbit condition, rather than just the physical location of the inducing stimuli.

**Figure 5:**
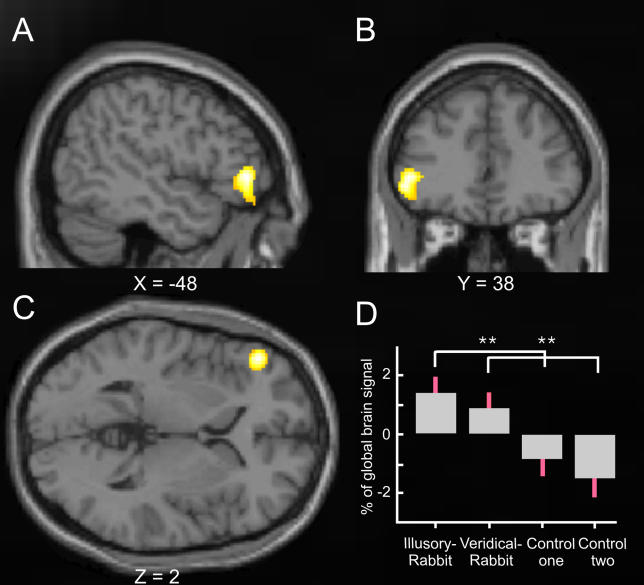
Common Activation for the Illusory-Rabbit and the Veridical-Rabbit versus Control Conditions beyond Somatosensory Cortex in the Whole-Brain Analysis Regions beyond the somatosensory cortex that displayed activity increases during both illusory- and veridical-rabbit, relative to control conditions (whole-brain, random-effects, group analysis). The graph shows the statistical T-map of the conjunction contrast of illusory-rabbit versus control data, and veridical-rabbit versus the other (equivalent but independent) control dataset (*p* < 0.001 for display). This revealed activation of the left inferior frontal gyrus (peak at X = −48, Y = 38, Z = 2). The format for the plot in (D) is as for the analogous plot inFigure 2D. Note that the veridical-rabbit and the illusory-rabbit conditions showed significantly higher activation than the two control datasets, which were equivalent, whereas the two rabbit conditions did not differ from each other.

In addition to hypothesis-driven analysis of somatosensory cortex, we also examined for completeness the whole brain volume for any further commonalities or differences in activity elicited by the experimental conditions. Beyond somatosensory cortex, both illusory- and veridical-rabbit stimulations commonly elicited stronger activity than the control condition in left inferior frontal gyrus (Figure 5). This region thus showed similar activity increases during the phenomenological percept of stimulation at site P2 for both the veridical- and the illusory-rabbit conditions, relative to the control condition, as had contralateral SI (see above). Our whole-brain analysis also revealed some regions that appeared more activated by the illusory rabbit than by its veridical counterpart. Specifically, two areas in right prefrontal (middle frontal gyrus) and right premotor (precentral/inferior frontal gyrus) cortex were significantly more active during the rabbit illusion than during the veridical-rabbit stimulation (seeFigure 6). Conversely, no regions were more active for the veridical than the illusory rabbit.

**Figure 6:**
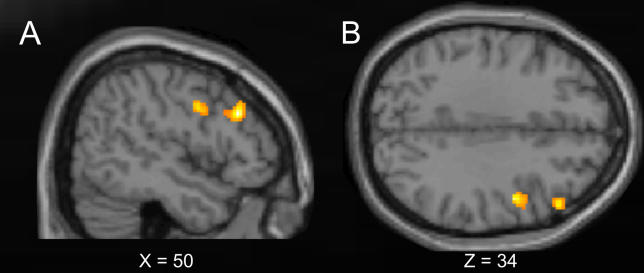
Activations for the Illusory-Rabbit versus the Veridical-Rabbit Conditions Regions beyond the somatosensory cortex that were more active for the illusory- than veridical-rabbit sequences (whole-brain, random-effects, group analysis). The graph shows the statistical T-map for the contrast illusory-rabbit minus veridical-rabbit, projected onto: (A) sagittal and (B) transversal slices of the Montreal Neurological Institute standard brain (*p* <0.001 for display purposes). This contrast revealed activation of the right dorsal prefrontal cortex (middle frontal gyrus, peak at X = 50, Y = 28, Z = 30) and of the right premotor cortex (precentral/inferior frontal gyrus, peak at X = 48, Y = 0, Z = 34).

## Discussion

We employed fMRI at 3T, with improved spatial resolution, to assess activity in somatosensory cortex during a version of the classic cutaneous rabbit illusion, as compared with sequences that actually stimulated the intervening skin site, and control sequences. Both the veridical-rabbit and the illusory-rabbit sequences elicited tactile sensations felt to correspond to the middle location (P2) along the forearm, which was stimulated in the veridical condition but not in the illusory condition. Critically, we were able to demonstrate that both these conditions commonly activated (relative to control sequences) a region in SI contralateral to the stimulated arm. Moreover, as confirmed by independent localizers, this activation fell in the appropriate somatotopic region for the intervening forearm site (P2) that was actually stimulated during the veridical-rabbit, but that was unstimulated yet still experienced phenomenally during the illusory-rabbit conditions (Figures 3 and4). No other region in somatosensory cortex was more activated for the two rabbit conditions than for the control condition, and indeed no somatosensory region differed between the two rabbit conditions. Peripheral adaptation or other stimulus-related effects, although potentially differing between the three experimental conditions, may thus seem unlikely to explain in any trivial manner the spatially specific activations corresponding to P2 somatotopy as found here. Taken together, these data suggest that the tactile illusion can affect human SI in a somatotopically appropriate manner, for the region that preferentially responds to the bodily location where the illusion was experienced (i.e., for the intervening skin site, to which hops of the illusory rabbit were mislocalized phenomenally, but which was not actually stimulated in the illusory condition).

There are some precedents for predicting that human SI might be involved in phenomenal tactile perception, as indicated here, but none make quite the same point as the present study. Electrical microstimulation of SI can elicit vibrotactile frequency discriminations in monkeys, comparable to those observed during physical peripheral stimulation [12]. Although this work sheds important light on neural codes for tactile properties [13], it investigated frequency rather than spatial somatotopic perception, unlike here, and did so in monkeys rather than humans. Recent human fMRI studies point to the possible involvement of SI in pathological tactile percepts (such as persistent touch [14], or phantom sensations [15,16] after amputation) as we now show here somatotopically for a normal illusion. A recent magnetoencephalography study [17] suggested that activity in somatotopic cortex might differ during an illusory shift in position of felt finger stimulation, but that study employed a cross-modal (rather than strictly somatosensory) illusion, and could measure somatotopic activity only via dipole localization/orientation, rather than with fMRI as here. Perhaps the most closely related prior study examined a different tactile mislocalization illusion, in anaesthetized monkeys rather than awake behaving humans [18]. In the funneling illusion, tactile mislocalization arises for brief stimuli presented simultaneously (rather than successively as here) at multiple locations. The pattern of activity revealed by optical imaging during anaesthesia in one region of monkey SI did not reflect the physical location of stimulation, but instead appeared consistent with the location that conscious human observers would have perceived under similar stimulus conditions [18]. The present study accords with this, but goes beyond it by directly relating conscious perception to somatotopic activity within human SI, in the same human participants at the same time, for the classic cutaneous rabbit illusion.

A computational model of early sensory cortex has been proposed that can accommodate the cutaneous rabbit illusion within a relatively simple dynamic neural network [19]. Our data support its proposal that spatiotemporal integration of stimuli, and the rabbit illusion in particular, can affect relatively early somatosensory areas in a dynamic manner, as for the activation of a somatotopically appropriate sector of SI found here (corresponding to P2), when stimulus sequences are manipulated at a sub-second level (with only the order of the same stimuli differing for illusory and control conditions here).

Anatomical terminology for different somatosensory regions has a less than straightforward history [20]. Here we have used the terms ‘primary somatosensory cortex' or ‘SI' in the conventional, generic sense of referring to somatosensory regions within the postcentral gyrus. Somatosensory cortex has been argued to have a hierarchical structure, according to which BA 3b might be regarded as ‘SI proper' [20]), receiving afferent input from peripheral cutaneous receptors [21]. On the hierarchical view, BA 1 of SI might be the next processing stage, with more complex receptive fields that integrate over larger spatial extents [21,22]. The activation observed here for both the illusory and the veridical rabbit (seeFigure 2) was tentatively suggested to lie predominantly within BA 1 of SI, according to a recent computerized probabilistic cytoarchitectonic map for interpreting human neuroimaging data [11]. But whatever its exact cytoarchitectonic attribution, this activity can be thought of as reflecting a processing stage involved in integrating somatosensory information from a spatially extended temporal pattern, yet still leading to a topographic pattern of activity. This would potentially correspond to layer 3 in the computational model of sensory dynamics [19].

Whereas the rabbit illusion reliably affected SI here, no activations specific to the rabbit reached significance for secondary somatosensory cortex. This nevertheless was activated by all three experimental conditions of tactile stimulation, as compared with the intervening passive-rest baseline (seeFigure S1). Those strong activations of secondary somatosensory cortex (SII) that were common to all three conditions, in the absence of reliable differential activity, may reflect the similar processing demands of our three stimulation conditions. Alternatively, it is conceivable that SII (and other areas) might in principle be affected by the rabbit illusion (as we establish here for SI), but in a manner not readily detectable with fMRI, perhaps because larger, more complex, or inter-digitated receptive fields [23] precluded any somatotopy from emerging in SII or other regions when using fMRI with the relatively subtle comparisons here.

We did find that one higher brain region, left inferior frontal cortex, was activated in common for the illusory- and veridical-rabbit conditions, relative to control (seeFigure 5). Tracing studies in monkeys have revealed some anatomical connections between this prefrontal area and SI [24]. Moreover, electrophysiological studies suggest further connectivity of these regions via secondary somatosensory and parietal cortex [25]. Although the functional impact of this prefrontal area for somatosensory processing remains largely unknown, activation there has been related to demanding somatosensory attention and working memory tasks in some previous studies [25–28], and might thus be implicated in similar aspects here.

Furthermore, two prefrontal areas beyond somatosensory cortex (precentral/inferior frontal gyrus and middle frontal gyrus) were activated by the rabbit illusion. Despite the phenomenologically similar percept, these regions showed higher blood oxygenation level-dependent activations for the illusory-rabbit than for the veridical-rabbit conditions, whereas no areas showed the reverse pattern. Activation of right prefrontal/premotor cortex has previously been found during various somatosensory-related cognitive tasks, including perceptual decision [29] in monkeys, tactile discrimination [30,31], crossmodal spatial and temporal integration of sensory information [32], and other bodily illusions [33]. We therefore speculate that these areas might be involved in top-down modulation of early somatosensory integrative processing (possibly via SII or posterior parietal cortex [34,35]). A recent study investigating a very different tactile illusion, known as the rubber hand illusion, also reported specific activation in these higher-level prefrontal areas [36].

Although previous, purely behavioral studies of the cutaneous rabbit illusion had led to speculations that it might involve SI [7], some other behavioral work had suggested that higher-level factors such as expectation [6] or crossmodal interactions [37] can sometimes be involved. At the level of neural populations, our human fMRI data now reveal that some higher-level prefrontal regions (precentral/inferior frontal gyrus and middle frontal gyrus) are indeed affected by the rabbit illusion, but more critically our data also show that SI is affected, in a somatotopically appropriate fashion. Just as recent human fMRI studies have shown that primary visual cortex may play a role in some aspects of conscious visual perception [1,2], our study indicates that this principle may also extend to the somatosensory domain.

In conclusion, by using fMRI during phenomenal reports of tactile perception, we were able to show that human SI is affected by the cutaneous rabbit illusion, leading to somatotopic activation of the felt (but not actually stimulated) body site. The intervening hops of the rabbit that get mislocalized and filled-in for conscious phenomenology evidently also get filled-in and appropriately re-localized within human SI.

## Materials and Methods

Thirteen healthy volunteers without history of neurological or psychiatric disease participated. Two were excluded as they did not reliably experience the tactile illusion, and one dataset was discarded because of technical problems during scanning. Eight of the remaining ten participants (mean age 24.8 y, range 19–34 y, 100% dextrality [38]) were naive to the purpose of the experiment. Written informed consent was obtained from each individual before investigation, in accord with local ethics clearance.

Prior to scanning, three pairs of surface-adhesive electrodes were positioned on the inner side of the left forearm of each individual, starting 3 cm from the wrist, and spaced by two equidistant gaps (mean = 9.3 ± 0.48 cm) in the direction of the elbow, to produce stimulation positions P1, P2, and P3 on the forearm (seeFigure 1). A constant current neurostimulator (DS7A, Digitimer, Hertfordshire, United Kingdom) was used to deliver electrical pulses (square wave, 2-ms duration) to the three sites. Electrode positions were adjusted individually until the participants did not feel any radiation of the stimulation away from each electrode. The sensory threshold for each site (P1 = 1.43 ± 0.28 mA; P2 = 1.24 ± 0.22 mA; P3 = 1.26 ± 0.34 mA) was determined using the method of limits [39]. During the experiment, the same constant, supra-threshold stimulus intensity (mean 4.37 ± 1.18 mA) was used for all sites, which did not elicit discomfort or any indications of muscle fasciculation.

Each participant underwent two practice sessions prior to scanning, one on the day before and one on the day of the main experiment, to minimize learning and habituation effects during the experiment. Inside the scanner, the three stimulus conditions (veridical-rabbit, illusory-rabbit, and control, see Introduction andFigure 1) were applied in random order, using a custom-built relay box, controlled by Matlab (The MathWorks, Natick, Massachusetts, United States) toolbox Cogent (http://www.fil.ion.ucl.ac.uk/cogent2000.html, running on a conventional computer. The inter-pulse interval for all trials and conditions was set to 50 ms, as this interval was found in pilot work to maximally induce the phenomenal rabbit illusion of apparent stimulation at position P2 (not physically stimulated) during P1-P1-P3 sequences (see also [5]). On veridical-rabbit trials, P1, P2, and P3 were stimulated successively with three pulses each. On illusory-rabbit trials, six stimulation pulses were delivered at P1, followed by three stimulation pulses at P3. Both rabbit conditions induced a similar percept of a pulse train, starting closely at the wrist and extending over the three electrode positions (Figure 1). Finally, in the control condition, three pulses were administered to P1, followed by three pulses to P3, and then three pulses to P1. No rabbit illusion was experienced under these circumstances, as expected. On each trial during scanning, a sequence of nine pulses was applied lasting 400 ms for each of the different conditions.

For the task, participants were instructed prior to the experiment to indicate on each trial by button press whether they felt stimulation to ‘include the middle electrode,' or instead stimulation ‘only of the outer electrodes.' They would thus be expected to make the former response on veridical-rabbit trials, and the latter response on control trials. For the illusory-rabbit trials, to the extent that these induced similar conscious perceptions as the veridical-rabbit trials, they should respond as if stimulation included the middle electrode (not included physically on such trials, but felt there if the rabbit illusion was induced). All responses and their latencies were acquired using a custom-built, scanner-compatible button box, recorded by the software used to deliver stimulation.

The experiment was performed with a 3T head scanner (Allegra, Siemens, Erlangen, Germany) using a standard head coil. The participant's head was immobilized with foam cushions to reduce movement. Functional images were acquired with a custom, improved-spatial-resolution sequence (single-shot gradient echo EPI) written specifically for this experiment to enhance our ability to detect functional somatotopy (matrix = 96 × 96, voxel size = 2 × 2 × 2 mm, flip angle = 90°, TE = 30 ms, TR = 2,890 ms, BW = 250 kHz, 50% spatial gap between adjacent slices) covering the whole cerebrum (34 slices). In addition, a structural T1-weighted image was acquired for each individual (matrix = 256 × 224, 176 slices, voxel size = 1 × 1 × 1 mm, TR = 7.92 ms, TE = 2.4 ms, TI = 910 ms, BW = 195 Hz/Px, flip angle = 15°). For stimulus presentation in the main experiment, we used an event-related design where the different conditions were randomly intermingled, with an inter-trial interval of 4–6 s between successive nine-pulse sequences of somatosensory stimulation for 400 ms. The hemodynamic response function was sampled every second, using jitter between the onset of stimulation and of volume acquisition. In order to minimize contamination of the somatosensory activations by response-related activations, the button press with either the right index or middle finger (to indicate whether stimulation was felt to include the middle electrode or not on the left forearm) was performed with the hand opposite to the stimulated left arm, and was delayed by over 1.5 s. Participants were then given a visual cue (written words ‘Respond now'), indicating the onset of the response interval. Note also that manual responses were modeled with a distinct regressor (see below) to partial out [40] any effects on neural activity in any case.

Three runs were performed for each participant, each run including 100 trials (260 image volumes) and lasting 12.5 min. In total, this resulted in data for each individual from 90 veridical-rabbit trials, 90 illusory-rabbit trials, and 120 control trials, which were acquired in randomly intermingled orders (see above). We presented more trials from the control condition, because these trials were then split in half (alternating control trials allocated to one or the other control dataset) to provide two independent control conditions with 60 trials each, for conjunction [10] analyses (see below).

In addition, we also performed a separate localizer run (230 image volumes, lasting 11 min) for each participant, in which we examined neural activations elicited by blocked stimulation of site P1, or P2, or P3 (using identical electrode positions as in the main experiment). In this run, each individual electrode site was repeatedly stimulated in a block design (inter-pulse-interval = 50 ms, pulse duration = 2 ms, length of stimulation block = 20 s, alternating with 20-s rest periods), with randomized order of the P1-, P2-, and P3-stimulation blocks.

Behavioral data were analyzed with paired t-tests using SPSS 11 (SPSS, Chicago, Illinois), and the fMRI analysis used SPM2 (http://www.fil.ion.ucl.ac.uk/spm). The first six images of each series were discarded from further analyses, to allow for stabilization of T1-effects. Pre-processing consisted of a slice-wise correction for acquisition delay to the middle slice, unwarping and realignment of the images, normalization to the Montreal Neurological Institute standard brain, plus spatial smoothing of the functional images with a three-dimensional Gaussian filter (FWHM 9 mm^3^). To assess whether blurring with such a filter might have affected our somatotopic results, we also repeated the same SPM analysis with a smaller Gaussian smoothing filter (FWHM 4 mm^3^), which led to the same results in SI (compare brain images inFigures 3 and4). We also used this minimal smoothing for the single-participant analyses (see below). Detrending of the whole dataset was performed with a linear model of the global signal [41]. Statistical parametric maps were calculated by multiple regression of the data onto a model of the hemodynamic response [40]. This model contained regressors for the onsets of correctly identified (see below) trials of the veridical-rabbit, illusory-rabbit, and control conditions, as derived by appropriately placed delta functions convolved with the canonical hemodynamic response function in SPM2. The control condition was split (alternatingly) into two regressors of 20 trials each per run, to provide independent baselines for the conjunction analysis [10]. In order to reduce error variance, two regressors separately coded either the trials with misclassified responses (seeProtocol S1 for post hoc fMRI analyses of these) or absent responses (together totalling 7% of the veridical-rabbit trials, 10% of the illusory-rabbit trials, and 13% of the control trials), or coded the motor response to all trials. The data were filtered with a 128-s cut-off, high-pass filter, and an AR(1)-model was used to account for serial correlation in the data. Commonalities and differences between conditions of interest were determined by random-effects analyses (t-tests) of the first-level contrast images.

The blocked localizer data were analyzed in a separate statistical model (but in identical anatomical space), containing 3 regressors for the onsets of the different stimulation sites (P1, P2, or P3), now derived from continuous series of delta functions (duration of 20 s) convolved with the canonical HRF. Cortical localizers for each of the three stimulation sites P1-P2-P3 were then derived (in group analyses) by random-effects comparisons of the differential contrast images of each stimulation site versus the mean of the two other sites. In addition, we used the individual localizer datasets (with reduced smoothing of 4-mm^3^ FWHM kernel) of every single participant to identify their own somatotopic peaks within right SI. Based on participant-specific somatotopic coordinates [P1 (X = 49.4 ± 1.8, Y = −17.2 ± 2.2, Z = 53.8 ± 2.8), P2 (X = 37.6 ±1.5, Y = −33.6 ± 2.4, Z = 62.6 ± 1.5), and P3 (X = 28.6 ± 2.8, Y = −35.4 ± 1.5, Z = 67.6 ± 1.6) mean and standard error], we then performed a single-participant analysis on the separate data of the main experiment (smoothed at 4-mm^3^ FWHM) by extracting the parameter estimates for each condition from a sphere of 2-mm radius, centered on the participant-specific somatotopic peaks from the localizers for P1, P2, or P3.

For analyses of activity in brain areas with a priori hypotheses (i.e., primary or secondary somatosensory areas), activations are reported at uncorrected significance level (*p* <0.001 uncorrected in SPM2), whereas all whole-brain analyses employed a significance level of*p* <0.05, corrected for multiple comparisons (family-wise error) across the brain volume.

## Supporting Information

Figure S1 Common Activation for the Stimulation Conditions Common activation for all stimulation conditions (i.e., illusory-rabbit, veridical-rabbit and control) in the main experiment relative to passive-rest baseline (whole-brain random-effects group analysis). Significant activations were observed for each stimulation condition in right SI, bilateral secondary somatosensory cortex, bilateral anterior insula, bilateral premotor cortex, and right thalamus (plus left sensorimotor cortex due to the button-press response).
(775 KB TIF).

Protocol S1 Post Hoc fMRI Analyses We performed a supplementary ROI analysis based on those rare experimental trials where stimulus sequences were not classified as expected behaviorally.
(24 KB DOC).

## Abbreviations

BA: Brodmann Area
fMRI: functional magnetic resonance imaging
FWHM: full-width half-maximum
ROI: region of interest
SI: primary somatosensory cortex
SII: secondary somatosensory cortex
T: tesla

## References

[pbio-0040069-b001] Rees G, Kreiman G, Koch C (2002). Neural correlates of consciousness in humans. Nat Rev Neurosci.

[pbio-0040069-b002] Ress D, Heeger DJ (2003). Neuronal correlates of perception in early visual cortex. Nat Neurosci.

[pbio-0040069-b003] Geldard FA, Sherrick CE (1972). The cutaneous “rabbit”: A perceptual illusion. Science.

[pbio-0040069-b004] Eagleman DM, Sejnowski TJ (2000). Motion integration and postdiction in visual awareness. Science.

[pbio-0040069-b005] Geldard FA (1975). Sensory saltation: Metastability in the perceptual world.

[pbio-0040069-b006] Kilgard MP, Merzenich MM (1995). Anticipated stimuli across skin. Nature.

[pbio-0040069-b007] Geldard FA, Sherrick CE (1983). The cutaneous saltatory area and presumed neural basis. Percept Psychophys.

[pbio-0040069-b008] Geldard FA (1982). Saltation in somesthesis. Psychol Bull.

[pbio-0040069-b009] Iwamura Y (2000). Bilateral receptive field neurons and callosal connections in the somatosensory cortex. Philos Trans R Soc Lond B Biol Sci.

[pbio-0040069-b010] Price CJ, Friston KJ (1997). Cognitive conjunction: A new approach to brain activation experiments. Neuroimage.

[pbio-0040069-b011] Eickhoff SB, Stephan KE, Mohlberg H, Grefkes C, Fink GR (2005). A new SPM toolbox for combining probabilistic cytoarchitectonic maps and functional imaging data. Neuroimage.

[pbio-0040069-b012] Romo R, Hernandez A, Zainos A, Salinas E (1998). Somatosensory discrimination based on cortical microstimulation. Nature.

[pbio-0040069-b013] Luna R, Hernandez A, Brody CD, Romo R (2005). Neural codes for perceptual discrimination in primary somatosensory cortex. Nat Neurosci.

[pbio-0040069-b014] Schwartz S, Assal F, Valenza N, Seghier ML, Vuilleumier P (2005). Illusory persistence of touch after right parietal damage: Neural correlates of tactile awareness. Brain.

[pbio-0040069-b015] Moore CI, Stern CE, Dunbar C, Kostyk SK, Gehi A (2000). Referred phantom sensations and cortical reorganization after spinal cord injury in humans. Proc Natl Acad Sci U S A.

[pbio-0040069-b016] Borsook D, Becerra L, Fishman S, Edwards A, Jennings CL (1998). Acute plasticity in the human somatosensory cortex following amputation. Neuroreport.

[pbio-0040069-b017] Schaefer M, Noennig N, Heinze HJ, Rotte M (2005). Fooling your feelings: Artificially induced referred sensations are linked to a modulation of the primary somatosensory cortex. Neuroimage.

[pbio-0040069-b018] Chen LM, Friedman RM, Roe AW (2003). Optical imaging of a tactile illusion in area 3b of the primary somatosensory cortex. Science.

[pbio-0040069-b019] Wiemer J, Spengler F, Joublin F, Stagge P, Wacquant S (2000). Learning cortical topography from spatiotemporal stimuli. Biol Cybern.

[pbio-0040069-b020] Kaas JH (1983). What, if anything, is SI? Organization of first somatosensory area of cortex. Physiol Rev.

[pbio-0040069-b021] Iwamura Y, Tanaka M, Sakamoto M, Hikosaka O (1993). Rostrocaudal gradients in the neuronal receptive field complexity in the finger region of the alert monkey's postcentral gyrus. Exp Brain Res.

[pbio-0040069-b022] Gardner EP, Costanzo RM (1980). Spatial integration of multiple-point stimuli in primary somatosensory cortical receptive fields of alert monkeys. J Neurophysiol.

[pbio-0040069-b023] Alloway KD, Burton H (1985). Homotypical ipsilateral cortical projections between somatosensory areas I and II in the cat. Neuroscience.

[pbio-0040069-b024] Preuss TM, Goldman-Rakic PS (1989). Connections of the ventral granular frontal cortex of macaques with perisylvian premotor and somatosensory areas: Anatomical evidence for somatic representation in primate frontal association cortex. J Comp Neurol.

[pbio-0040069-b025] Hagen MC, Zald DH, Thornton TA, Pardo JV (2002). Somatosensory processing in the human inferior prefrontal cortex. J Neurophysiol.

[pbio-0040069-b026] Romo R, Brody CD, Hernandez A, Lemus L (1999). Neuronal correlates of parametric working memory in the prefrontal cortex. Nature.

[pbio-0040069-b027] Passingham RE, Ettlinger G (1972). Tactile discrimination learning after selective prefrontal ablations in monkeys (Macaca mulatta). Neuropsychologia.

[pbio-0040069-b028] Roland PE (1987). Somatosensory detection in patients with circumscribed lesions of the brain. Exp Brain Res.

[pbio-0040069-b029] Romo R, Hernandez A, Zainos A (2004). Neuronal correlates of a perceptual decision in ventral premotor cortex. Neuron.

[pbio-0040069-b030] Stoeckel MC, Weder B, Binkofski F, Buccino G, Shah NJ (2003). A fronto-parietal circuit for tactile object discrimination: An event-related fMRI study. Neuroimage.

[pbio-0040069-b031] Harada T, Saito DN, Kashikura K, Sato T, Yonekura Y (2004). Asymmetrical neural substrates of tactile discrimination in humans: A functional magnetic resonance imaging study. J Neurosci.

[pbio-0040069-b032] Fuster JM (2001). The prefrontal cortex–an update: Time is of the essence. Neuron.

[pbio-0040069-b033] Fink GR, Marshall JC, Halligan PW, Frith CD, Driver J (1999). The neural consequences of conflict between intention and the senses. Brain.

[pbio-0040069-b034] Petrides M, Pandya DN (1984). Projections to the frontal cortex from the posterior parietal region in the rhesus monkey. J Comp Neurol.

[pbio-0040069-b035] Rizzolatti G, Luppino G (2001). The cortical motor system. Neuron.

[pbio-0040069-b036] Ehrsson HH, Spence C, Passingham RE (2004). That's my hand! Activity in premotor cortex reflects feeling of ownership of a limb. Science.

[pbio-0040069-b037] Kamitani Y, Shimojo S (2005). Sound-induced visual 'rabbit'. J Vis.

[pbio-0040069-b038] Oldfield RC (1971). The assessment and analysis of handedness: The Edinburgh inventory. Neuropsychologia.

[pbio-0040069-b039] Fechner GT (1860). Elemente der Psychophysik.

[pbio-0040069-b040] Friston KJ, Holmes AP, Poline JB, Grasby PJ, Williams SC (1995). Analysis of fMRI time-series revisited. Neuroimage.

[pbio-0040069-b041] Macey PM, Macey KE, Kumar R, Harper RM (2004). A method for removal of global effects from fMRI time series. Neuroimage.

