# Supplementary material for: The Cutaneous Rabbit Illusion Affects Human Primary Sensory Cortex Somatotopically
Source: PLoS Biol. 2006 Feb 28;4(3):e69. doi: 10.1371/journal.pbio.0040069 (PMC1382015; doi:10.1371/journal.pbio.0040069)
Supplement: Supplementary file 1 [file pbio.0040069.sd001.doc]

Supporting text:

As the suggestion of a referee, we also performed a supplementary ROI analysis based on those rare experimental trials where stimulus sequences were not classified as expected behaviorally (i.e. P2 reported as not stimulated although it was during 5.7% of veridical rabbit trials overall; illusion at P2 not experienced during 6.5% of illusory rabbit trials; and P2 reported during 6.1% of control trials). Note that occasional failures to respond at all on a single trial (3.9% of trials overall) were not included as misclassifications in this particular analysis. Moreover, three subjects never made misclassifications, so could not be included in post hoc fMRI analysis of these. For the remaining subjects, activity in the cortical representation of P2 (determined individually, as described in the main text) was higher (p<.05) during misclassification of control trials (where subjects mistakenly reported P2 stimulation) than during those rare veridical- or illusory-rabbit trials where P2 was reported as not felt.

Although we do not base strong conclusions on this post hoc comparison (since we did not plan it, and had only limited power to assess it given the rarity of behavioral responses that were not as expected), we note that it further accords with our main conclusion from the illusion-related activations (Figs. 2-4 in main paper); namely, that somatotopic activations in S1 can relate more closely to perceived location than to stimulus location per se.
